# Application of the Metal Reflector for Redistributing the Focusing Intensity of SPPs

**DOI:** 10.3390/nano10050937

**Published:** 2020-05-13

**Authors:** Jiaxin Ji, Pengfei Xu, Zhongwen Lin, Jiying Chen, Jing Li, Yonggang Meng

**Affiliations:** 1 College of Mechanical and Electronic Engineering, China University of Petroleum, Qingdao 266580, China; xupfupc@163.com (P.X.); linzhongwen_upc@163.com (Z.L.); lijing85@upc.edu.cn (J.L.); 2 School of Mechanical Engineering, Dalian University of Technology, Dalian 116024, China; 3 College of New Energy, China University of Petroleum, Qingdao 266580, China; Ying1606050115@163.com; 4 State Key Laboratory of Tribology, Tsinghua University, Beijing 100084, China; mengyg@tsinghua.edu.cn

**Keywords:** metal reflector, energy flux density, energy redistribution, rotational near-field photolithography

## Abstract

The near-field photolithography system has attracted increasing attention in the micro- and nano-manufacturing field, due to the high efficiency, high resolution, and the low cost of the scheme. Nevertheless, the low quality of the nano-patterns significantly limits the industrial application of this technology. Theoretical calculations showed that the reason for the poor nano-patterns is the sharp attenuation of the surface plasmon polaritons (SPPs) in the photoresist layer. The calculation results suggest that the waveguide mode, which is composed of the chromium-equivalent dielectric layer-aluminum, can facilitate the energy flux density distribution in the photoresist layer, resulting in the enhancement of the field intensity of SPPs in the photoresist layer. This reduces the linewidth of nano-patterns, while it enhances the pattern steepness. Eventually, the focusing energy of the photoresist layer can be improved. The finite-difference time-domain method was employed to simulate and verify the theoretical results. It is found that for the rotational near-field photolithography with 355 nm laser illumination, the linewidths of the nano-patterns with and without the aluminum reflector are 17.54 nm and 65.51 nm, respectively. The robustness of the experimental results implies that the application of the aluminum reflector enhances the focusing effect in the photoresist, which can broaden the application of the near-field photolithography.

## 1. Introduction

**Figure 1:**
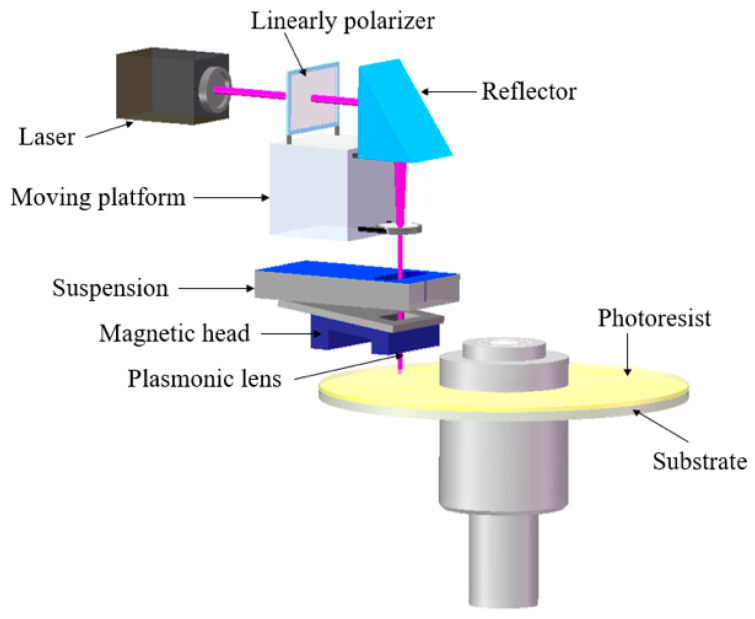
Schematic configuration of the rotational near-field photolithography.

Near-field lithography is a technology far beyond the diffraction limit nano-patterning that is applied to exploit diffracted fields, including quasi-spherical waves and surface plasmon polaritons (SPPs) [1,2,3,4,5,6,7,8,9,10]. These waves are radiated by a grating or prism, that can help the incident light confined to a super small scale through the strong near-field coupling via evanescent photons [11]. Studies show that the radiation energy generated from the strong coupling in the horizontal direction can be applied to expose the near-field photoresist. The rotational near-field photolithography (RNFP) technology emerged to guarantee the performance of SPPs at an effective distance and a high throughput [12]. Based on the working principle of the hard disk drive, it was found that the aerodynamics between the head and the disk provide a stable working distance of less than 20 nm [13]. Figure 1 schematically shows the RNFP system. The air bearing force between the head and the disk is instrumental to realizing the near-field working distance of 10 nm. The designed plasmonic lens to excite SPPs was fabricated at the tail of the head. The coated photoresist on the disk surface was exposed by a high-energy focused beam of SPPs. Moreover, the RNFP resolution was successfully demonstrated with a below 30 nm half-pitch as an alternative low-cost nanofabrication [14,15]. Since the hard disk drive operates at 10 m/s, the RNFP requires only 5 min to process a 12-inch disk. Both the width and depth-of-field of the nano-patterns are key indicators to evaluate the processing outcomes. Researchers applied the transfer-matrix theory, higher numerical aperture, and effective-gain-medium resonance to optimize the resonance wavelength of SPPs and obtained high depth-of-field nanostructures [16,17,18,19,20]. However, reviewing the literature indicates that the foregoing investigations suffer from a critical issue in terms of the linewidth and the steepness of the nano-pattern, which can limit the applications of the RNFP.

The linewidth, depth, and the steepness of nano-patterns are key indicators of the development quality. Hence, it is necessary to ensure the penetrating radiation of SPPs in the photoresist layer and the consistency of the focusing effect at the entrance and outlet of the photoresist layer. In this regard, SPPs should achieve a strong and consistent energy distribution in the photoresist layer. However, the propagation characteristics of SPPs are in contrast with the developing requirements. In fact, the radiation energy decreases exponentially as the vertical distance increases [21]. Therefore, maintaining a uniform and strong field enhancement effect in the photoresist layer is of significant importance to increase the field depth, reduce the line edge roughness, and improve the development quality. Researchers [22,23,24,25,26] showed that the superlenses and the waveguide can effectively improve the steepness and the focal depth of patterns through the reflection. Hence, the metal-insulator-metal (MIM) waveguide mode of the chromium, the plasmonic lens machined substrate, the equivalent dielectric layer (the air-photoresist layer) and the metal reflective layer are formed by constructing metal reflective layer on the surface of the quartz disc. This paper intends to evaluate the field confinement ability of the MIM waveguide to improve the distribution of the optical field energy in each film layer.

## 2. Optimization of the Energy Flux Density in the Photoresist Layer

**Figure 2:**
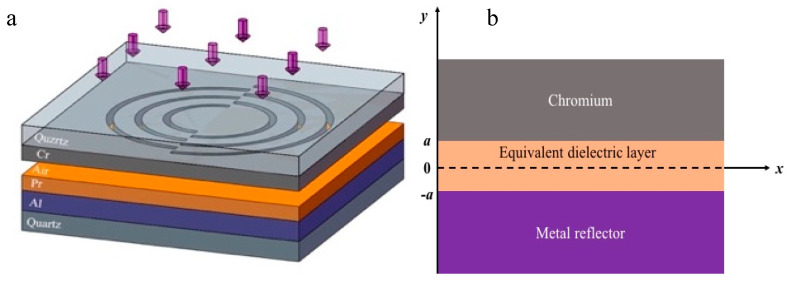
Configuration of the metal-insulator-metal (MIM) heterostructure in the rotational near-field photolithography (RNFP) system (**a**), and its longitudinal section (**b**).

As a metal heterostructure, the MIM waveguide can restrict the optical field energy through different absorption characteristics of each layer so that the focusing effect on the corresponding sub-wavelength scale can be investigated. By constructing the MIM waveguide in the etched structure, the appearance of the radiation mode and the leakage mode can be avoided. Consequently, the energy flux density of SPPs in the photoresist can be improved. Figure 2a illustrates the diagram of the MIM heterostructure in the RNFP system. Investigations indicated that chromium has remarkable mechanical strength and chemical stability, which can be utilized to process the plasmonic lens to excite SPPs under 355 nm illumination [27]. Therefore, a chromium layer with 60 nm thickness is used at the photolithography head for the RNFP system. The findings show that the equivalent medium method can be applied to equalize the air and the photoresist layers. The aluminum film is regarded as the metal reflector. Therefore, chromium-equivalent dielectric layer-aluminum constitutes the basic MIM waveguide. Compensating the intensity attenuation of the vertical field with the metal reflector is an effective method to improve the steepness and resolution of the development pattern, based on the principle of MIM waveguide. However, the dielectric constant of the metal reflector significantly affects the propagation characteristics of SPPs, resulting in different distributions of the energy flux density in the photoresist layer. In light of this, the material selection of the metal substrate is the key factor to determine the internal energy intensity of the photoresist layer. In order to facilitate the theoretical analyses, the equivalent transformation is carried out to transform the model shown in Figure 2a to that in Figure 2b. The origin point and the *x*-axis represent the center of the equivalent dielectric layer and the horizontal direction of the interface, respectively. Moreover, the distance between “−a” to “a” represents the total vertical length of the equivalent dielectric layer. Figure 2b indicates that the chromium layer and the metal reflective layer are located beyond “a” and behind “−a” of the *y*-axis, respectively.

The intensity and distribution of the focusing energy in the photoresist layer characterizes the development pattern quality. Moreover, the distribution effect can be described by the energy flux density of the electromagnetic field in the vertical direction, which is called the vertical component of the Poynting vector [28,29,30,31,32,33,34,35,36,37]:

(1) $$S_{z,i}=E_{x}H_{y}$$

where *S_z_*_,*i*_ denotes the energy flux density of the *i*th layer of the MIM waveguide in the z-direction. Furthermore, *H_y_* and *E_x_* denote the magnetic field intensity in the *y*-direction and the electric field intensity in the *x*-direction, respectively. As the SPPs propagating along the metal/dielectric interface can only be excited by the transverse magnetic (TM) wave illumination, the electromagnetic wave should satisfy the magnetic field continuity in the *y*-direction and the electric field continuity in the *x*- and *z*-direction [21]. Since the difference between dielectric constants of each MIM film affects the amplitude and the electromagnetic field characteristics, the definitions of the *H_y_* term in the metal and dielectric layers are slightly different. The field equation for exciting SPPs in the chromium metal layer can be described by Equations (2)–(4). Moreover, Equations (5)–(7) and (8)–(10) describe the field equation in the equivalent dielectric layer and the metal reflector, respectively.

(2) $$H_{y}=Ae^{i\beta x}e^{-k_{1}z}$$

(3) $$E_{z}=-A\frac{\beta }{\omega \varepsilon_{0}\varepsilon_{1}}e^{i\beta x}e^{-k_{1}z}$$

(4) $$E_{z}=-A\frac{\beta }{\omega \varepsilon_{0}\varepsilon_{1}}e^{i\beta x}e^{-k_{1}z}$$

(5) $$H_{y}=Be^{i\beta x}e^{k_{2}z}+Ce^{i\beta x}e^{-k_{2}z}$$

(6) $$E_{x}=-iB\frac{1}{\omega \varepsilon_{0}\varepsilon_{2}}k_{2}e^{i\beta x}e^{k_{2}z}+iC\frac{1}{\omega \varepsilon_{0}\varepsilon_{2}}k_{2}e^{i\beta x}e^{-k_{2}z}$$

(7) $$E_{z}=B\frac{\beta }{\omega \varepsilon_{0}\varepsilon_{2}}e^{i\beta x}e^{k_{2}z}+C\frac{\beta }{\omega \varepsilon_{0}\varepsilon_{2}}e^{i\beta x}e^{-k_{2}z}$$

(8) $$H_{y}=De^{i\beta x}e^{k_{3}z}$$

(9) $$E_{x}=-iD\frac{1}{\omega \varepsilon_{0}\varepsilon_{3}}k_{3}e^{i\beta x}e^{k_{3}z}$$

(10) $$E_{z}=-D\frac{\beta }{\omega \varepsilon_{0}\varepsilon_{3}}e^{i\beta x}e^{k_{3}z}$$

In these equations, *A*, *B*, *C,* and *D* denote the amplitude. Moreover $k_{1}$, $k_{2}$ and $k_{3}$ represent the propagation vectors of SPPs in the chromium layer, the equivalent dielectric layer, and the metal reflection layer, respectively. On the other hand, β, ω, $\varepsilon_{0}$ and $\varepsilon_{i}$ are the propagation constants of SPPs, propagation frequency of the light, dielectric constant of the air, and the dielectric constant of the material in the *i*th layer, respectively. Introducing the electric field intensity *E_x_* in the MIM three layers, (1) for the vertical component of the Poynting vector can be simplified in the form below:

(11) $$S_{z,i}=\frac{1}{2}\frac{\beta }{\omega \varepsilon_{0}\varepsilon_{i}}H_{y}^{2}$$

Since ω, $\varepsilon_{0}$, $\varepsilon_{i}$ and $k_{i}$ are constants in each layer, the distribution of *H_y_* determines the distribution of the Poynting vector in each layer so that the y-direction can also be characterized in the z-direction. Since ultraviolet light with a wavelength of 355 nm is used as the illuminant, the precious metals, including chromium, aluminum, and silver, should be selected to excite SPPs. In this wave band, the dielectric constants of chromium, aluminum, silver, air, and photoresist are −8.3435+8.4588i, −18.3418+3.311i, −2.0299+0.60192i, 1 and 2.455, respectively [38]. In the MIM model, the dielectric constant of the equivalent dielectric layer, which is composed of the photoresist and air, is equivalent to the following expression: $\varepsilon =\sum \varepsilon_{i}h_{i}/\sum h_{i}$, where the calculated value is 2.091. In order to determine the correlation between wave vectors $k_{i}$ and β, known parameters should be introduced into the wave Equation (12). Then parameter β can be calculated from (13) so that $k_{i}$ can be obtained [20,39] when the thickness of the air and photoresist layers are 10 and 30 nm, respectively. In this case, the parameter “*a*” in Figure 2b is assumed to be 20 nm.

(12) $$k_{i}^{2}=\beta^{2}-k_{0}^{2}\varepsilon_{i}$$

(13) $$e^{-4k_{1}a}=\frac{k_{1}/\varepsilon_{1}+k_{2}/\varepsilon_{2}}{k_{1}/\varepsilon_{1}-k_{2}/\varepsilon_{2}}\frac{k_{1}/\varepsilon_{1}+k_{3}/\varepsilon_{3}}{k_{1}/\varepsilon_{1}-k_{3}/\varepsilon_{3}}$$

The amplitude *A*, *B*, *C,* and *D* in each layer must be obtained to determine the distribution of *H_y_* in the photoresist and to analyze the distribution effect of the focusing energy in each layer. These amplitudes can be calculated according to the propagation characteristics of SPPs and the continuation theorem. Equations (14) and (15) can be obtained from the continuity of *H_y_* and *E_x_* at the interface between the equivalent layer and the metal reflector. While Equations (16) and (17) can be obtained from the continuity of *H_y_* and *E_x_* at the interface between the chromium layer and the equivalent layer.

(14) $$De^{-k_{3}a}=Be^{-k_{2}a}+Ce^{k_{2}a}$$

(15) $$-\frac{D}{\varepsilon_{3}}k_{3}e^{-k_{3}a}=-\frac{B}{\varepsilon_{2}}k_{2}e^{-k_{2}a}+\frac{C}{\varepsilon_{2}}k_{2}e^{k_{2}a}$$

(16) $$Ae^{-k_{1}a}=Be^{k_{2}a}+Ce^{-k_{2}a}$$

(17) $$\frac{A}{\varepsilon_{1}}k_{3}e^{-k_{1}a}=-\frac{B}{\varepsilon_{2}}k_{2}e^{k_{2}a}+\frac{C}{\varepsilon_{2}}k_{2}e^{-k_{2}a}$$

**Figure 3:**
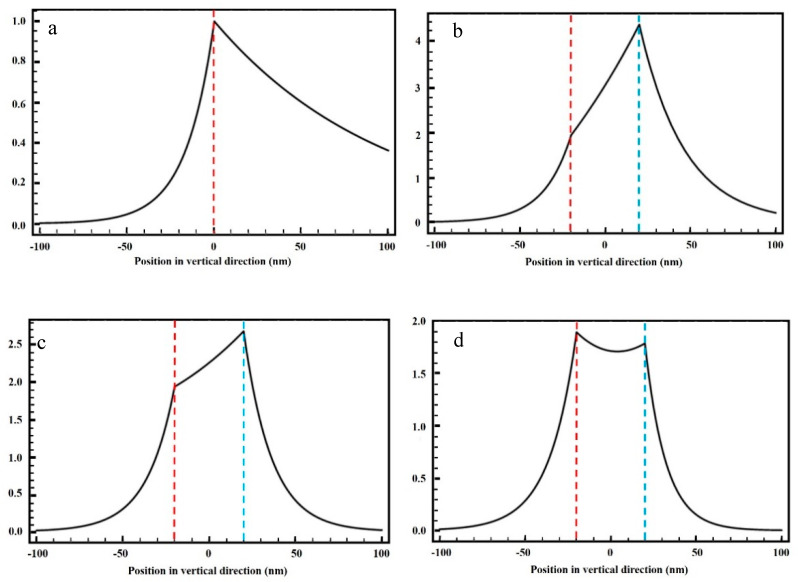
Surface plasmon polaritons (SPPs) distribution of the dimensionless *H_y_* component in the photoresist layer with different materials. (**a**) No metal reflector, (**b**) silver reflector, (**c**) chromium reflector, and (**d**) aluminum reflector.

Correlations among amplitudes *A*, *B*, *C,* and *D* in each dielectric layer can be obtained through the characteristic solutions for Equations (14) to (17). Introducing the parameters obtained in the previous part into Equations (2), (5), and (8), the energy flux distribution of the magnetic field can be obtained with the film in the central position of the excitation SPPs structure for different reflective film materials. Figure 3 indicates that the variation of the magnetic field intensity *H_y_* can characterize the distribution of the energy flux density *S_z_* in accordance with Equation (11). In other words, Figure 3 illustrates the distribution trend of the energy flux density in each film layer.

The longitudinal coordinate in Figure 3 represents the dimensionless magnetic field components, while the abscissa represents the position variation in each vertical direction, which was previously illustrated as the *y*-axis in Figure 2b. The red dotted line is the interface between the chromium (the left part) and the equivalent layer (the right part), where SPPs are excited. Moreover, the blue dotted line is the interface between the equivalent layer (the left side) and the metal reflector (the right side). The equivalent dielectric layer between the two dotted lines is the developing layer, which is the main observation section of the energy distribution. Figure 3a shows the distribution of *H_y_* when there is no metal reflector. It can be observed that the field intensity of SPPs excited by the interface of the chromium-equivalent layer decreases rapidly as the distance between the chromium film and the photoresist increases. When the light beam energy approaches the SPPs excitation level, it can reach the exposure threshold of the photoresist. Then the energy continues to decay rapidly after reaching the photoresist interface, which is very harmful to the linewidth, depth, and the steepness of the RNFP system. Figure 3b,c illustrate the silver and chromium reflective layers, respectively. The findings show that adding the metal reflector remarkably improves the energy distribution of SPPs in the developing layer. Furthermore, Figure 3b,c indicate that the energy flux distribution at the interface between the equivalent layer and the reflective layer is the highest, which greatly improves the energy distribution in the photoresist layer, and simultaneously improves the development depth. However, it is observed that the energy distribution in the equivalent dielectric layer is inconsistent with the two interfaces, which adversely affects the linewidth resolution and the steepness of the processed graphics. When the aluminum reflector is utilized, the energy distribution at the two interfaces slightly changes. Figure 3d shows that the SPPs reflected by the aluminum film are reasonably coupled with the SPPs excited by the chromium-air interface. The uniform distribution of the field intensity of SPPs in the photoresist layer improves the depth and the steepness of the near-field lithography, and it ensures high resolution and consistency of the processing linewidth. Considering all foregoing factors, the aluminum reflector is the preferred reflector in the RNFP system.

**Figure 4:**
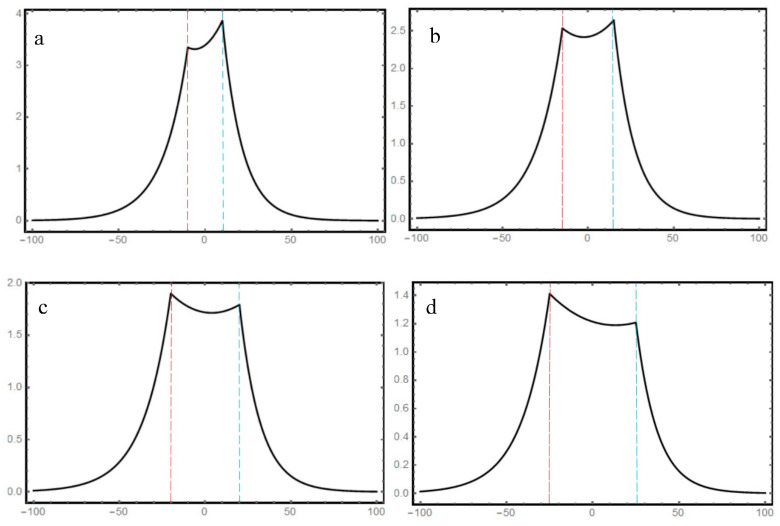
SPPs’ distribution of the dimensionless H_y_ component in the photoresist layer with different equivalent dielectric layer thicknesses. (**a**–**d**) correspond to the thicknesses of 30, 40, 50, and 60 nm, respectively.

While the thickness of the equivalent dielectric layer is affected by the flying height and the photoresist thickness. During the photolithography, the flying height between the head and disk changes within the range of 10–15 nm. Meanwhile, the thickness of the photoresist prepared by the spin-coating method also fluctuates within the range of 30–35 nm due to the system error. The variations of flying height and photoresist thickness lead to changes in the dielectric thickness, this affects the energy flux density distribution of the MIM heterostructure. Figure 4 shows the SPPs distribution in MIM as the thickness of the dielectric film changes gradually. It can be observed that following the energy conservation law, the intensity of H_y_ decreases as the equivalent dielectric film thickness gradually increases. However, the energy flux has little variation and is still uniformly distributed in the dielectric film. This indicates that the energy distribution is not sensitive to the change of the dielectric thickness.

## 3. Simulation Results and Discussions

**Figure 5:**
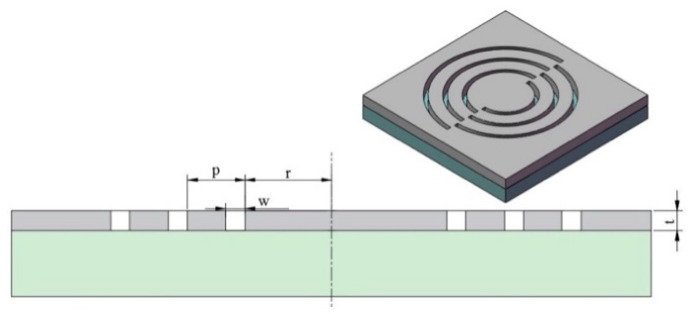
Schematic and parameters of the designed plasmonic lens.

Based on the transfer matrix theory, the resonance wavelength of SPPs in the RNFP system with a non-metallic reflector structure is about 240 nm [20]. On the other hand, employing the same method with the aluminum reflector structure proves that the resonance wavelength reduces to 200 nm. Therefore, the plasmonic lens with a periodic semi-circular structure is designed. Figure 5 shows the configuration of the designed structure. Moreover, the corresponding specific parameters are shown in Table 1.

In Figure 5, *p*, *r* and *w* are the groove pitch, the width of the first ring, and the groove width, respectively. Moreover, t and n_u_ denote the chromium layer thickness and the groove number, respectively. It should be noted that the optimization methods of each parameter are discussed in detail in reference [16].

**Figure 6:**
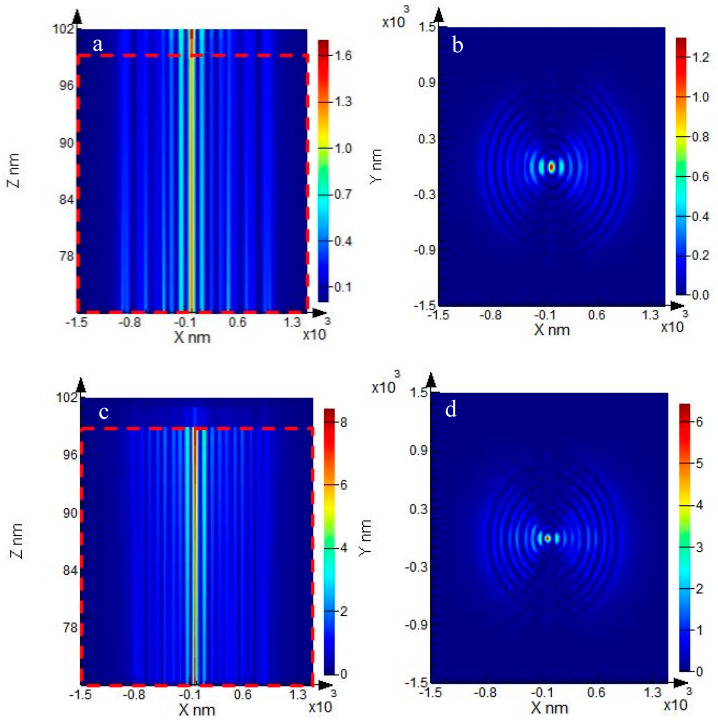
Results from the FDTD Solutions software, (**a**) with no metal reflector and (**c**) with the aluminum reflector. (**b**) and (**d**) are the cross-section of (**a**) and (**c**) at 85 nm, respectively.

Figure 6 shows the obtained results, which were verified through the FDTD Solutions software. Figure 6a illustrates the energy distribution of SPPs focusing effect, while no metallic reflector is installed. It presents the photoresist layer within the red dotted line frame (70–100 nm), while the quartz substrate is located beyond 100 nm. Figure 6b shows the cross-section of the spot at 85 nm. Figure 6c shows the energy distribution of SPPs for the most used metal reflectors on aluminum substrates. Similarly, the photoresist layer is located within the red dotted line frame (70–100 nm), while the metal reflector layer is located beyond 100 nm. Figure 6d shows the cross-section of the spot at 85 nm, which is much smaller than that of Figure 6b. Moreover, Figure 6a indicates that the focusing energy of the exit layer (100 nm) is lower than that of the entrance layer (70 nm). Furthermore, it is found that the maximum field enhancement factor and the diameter of the focusing spot are 1.7 and 50 nm, respectively. A comparison of Figure 6a,c indicates that the application of aluminum reflector not only reduces the energy attenuation of SPPs at the interface of the photoresist entry but it also effectively improves the field enhancement factor of SPPs in the photoresist layer. Figure 6b,d show that the optimal full width at half maximum (FWHM) can reach 25 nm indicating that the MIM waveguide structure can substantially reduce the beam spot diameter. Moreover, Figure 3 and Figure 6 indicate that the aluminum reflector increases the energy distribution of SPPs in the equivalent dielectric layer, thereby reducing the beam spot diameter. Similar values of the energy entering and leaving the photoresist interface demonstrate that the structure is beneficial for improving the steepness of the near-field photolithography system. The obtained simulation results are consistent with the theoretical analysis results.

## 4. Experimental Results and Discussion

**Figure 7:**
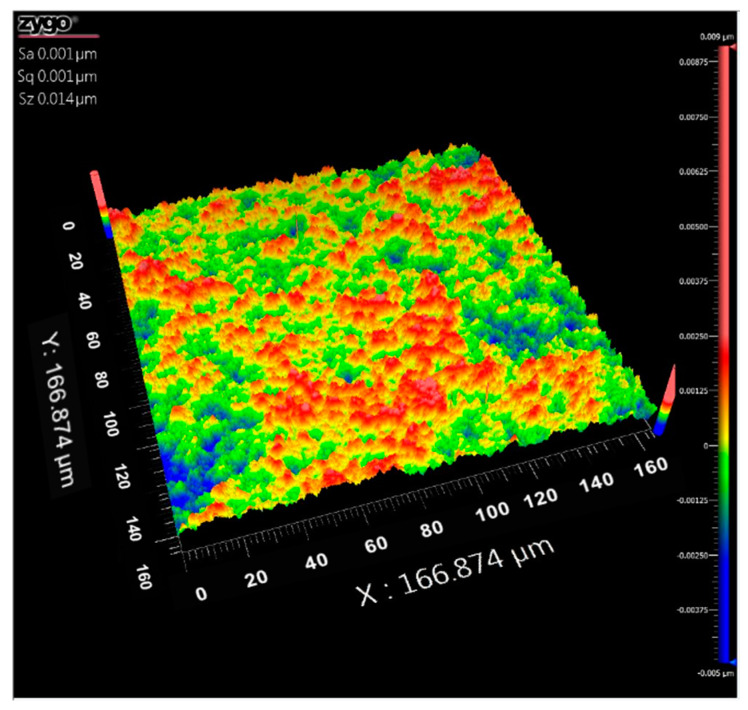
The morphology of the sputtered aluminum film measured by the white-light interferometer.

The aluminum film with a thickness of 80 nm was prepared on the quartz disc surface by the sputtering, where the MSP-300B sputtering coating instrument is applied in this regard. Furthermore, the sputtering power, pressure, and time were set to 300 W, 0.2 Pa, and 50 s, respectively. Figure 7 shows the film morphology measured by the white-light interferometer. The average surface roughness is 1 nm without apparent defects demonstrating that the flatness of the aluminum substrate is beneficial to the stable flight of the lithography head. Cee 200X desktop homogenizer at 3000 rpm is employed to prepare the self-made photoresist FPT-8Boc in accordance with the requirements of reference [15]. The solvent is completely removed by applying the Cee 1300X drying table for 180 s after drying at the sputtered film FPT-8Boc photoresist for 90 s. Eventually, a photoresist film thickness of 30 nm is obtained.

**Figure 8:**
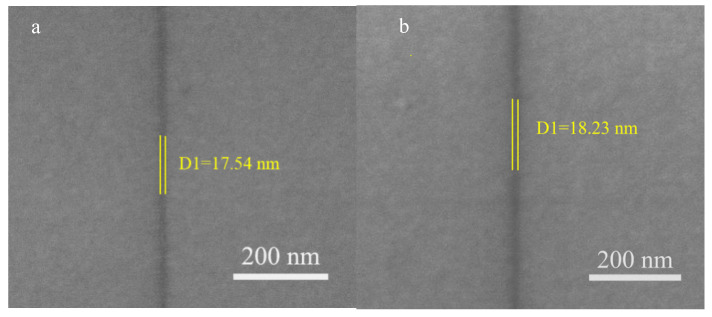

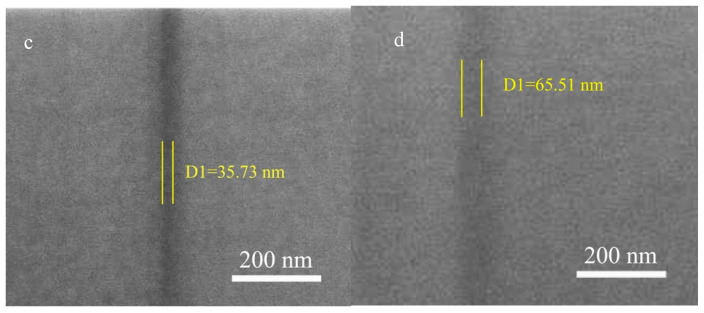
SEM scanning of RNFP development results. (**a**,**b**) are development results for the aluminum reflector, (**c**) is the development result for the chromium reflector, (**d**) is etching result without the aluminum reflector.

The loading mode of the flight transition zone is utilized to ensure the stable flight of the etching head of the plasmonic lens on the photoresist surface [13,40]. Figure 8 shows the obtained development results from the SEM scanning with 50 mW laser irradiation. It should be noted that the FIB/SEM/GIS integrated mode dual-beam focused ion beam system of TESCAN Company is applied for the SEM photography. Prior to the scanning, a platinum film with a thickness of 1 nm is deposited on the photoresist surface. Moreover, the corresponding beam current is set to 15 kV. The magnification factor is 200,000 times. Figure 8a shows the development linewidth is less than 20 nm for the aluminum reflector. It is worth noting that the same processing methods as those of Figure 8a are applied to interpret Figure 8b. However, the aluminum film thickness is slightly different in these two figures. This difference originates from the difference in the sputtering time. Similar linewidths indicate that the development results are robust to the thickness change of the aluminum film. Moreover, Figure 8c shows the development result for the chromium reflector. The wider linewidth in Figure 8c than those of Figure 8a,b indicates that the reflection property of the chromium is weaker than that of the aluminum reflector. These development results are consistent with the simulation results observed in Figure 3. The brilliant linearity roughness also proves the effectiveness of the energy coupling of metal reflectors. Comparing the results obtained with those of the 65.51 nm linewidth using the non-metallic reflector (Figure 8d) indicates the important role of the energy density optimization in the developed photoresist. The development results show that the processing system has reasonable robustness to the etching structure, ensuring the suitability of the RNFP system in industrial applications.

## 5. Conclusions

A high light intensity distribution and consistency in the photoresist layer guarantee a low etching linewidth, high etching depth, and a reasonable steepness of the nano-patterns. Theoretical calculations show that the energy density of each layer in the MIM waveguide structure is mainly affected by the material of the metal reflector. When the aluminum reflector is applied, higher intensity distribution can be obtained in the photoresist layer. In the present study, the finite-difference time-domain method is applied to verify the accuracy of theoretical results. The findings show that the application of the aluminum reflective film increases the maximum enhancement factor to more than 8, while the beam spot diameter decreases to 25 nm. This demonstrates that the MIM waveguide structure, which is composed of the chromium-equivalent dielectric layer-aluminum, is indeed helpful for improving the energy density in the photoresist. The matrix transfer theory was applied to calculate the propagation wavelength of SPPs in the MIM waveguide structure, where the half-ring structure calculation of the plasmonic lens has a period of 100 nm. In this regard, the plasmonic lens is fabricated for RNFP experiments. The experimental results show that the application of the aluminum reflective film reduces the linewidth to 17.54 nm, which is superior to 65.51 nm for the conventional scheme without the reflector. Moreover, the findings demonstrate that the aluminum reflector is useful for improving the focusing effect of the photoresist. The robustness of the developmental results in the thickness variation of the aluminum film is also conducive to the RNFP system, stimulating the development of advanced electronics of the future.

## Figures and Tables

**Table 1:** Optimized structural parameters of the plasmonic lens.

Parameters	*p* (nm)	*r* (nm)	*w* (nm)	*t* (nm)	*n_u_* (nm)
200 nm peridor	200	300	100	60	6
240 nm peridor	240	360	100	60	66
